# Reply to Lombardo, 2020: An additional route of investigation: what are the mechanisms controlling ribosomal protein genes dysregulation in autistic neuronal cells?

**DOI:** 10.1038/s41380-020-0792-7

**Published:** 2020-05-28

**Authors:** Karina Griesi-Oliveira, Maria Rita Passos-Bueno

**Affiliations:** 1 Hospital Israelita Albert Einstein, São Paulo, Brazil; 2 Departamento de Genética e Biologia Evolutiva, Instituto de Biociências, Universidade de São Paulo, São Paulo, Brazil

**Keywords:** Genetics, Neuroscience

## To the Editor:

We have recently reported our findings relative to a transcriptome study in iPSC-derived neuronal cells from autistic patients, which revealed the dysregulation of a co-expression module of synaptic genes in neurons (M_Neur_1-turquoise) and of a module of genes related to translation in neuronal progenitor cells (M_NPC_10-blue) from these patients [1]. Comparison of our data to other similar selected studies revealed that the module of synaptic genes has been consistently replicated in the literature as dysregulated in ASD neurons. On the other hand, we did not find evidence of dysregulation of M_NPC_10-blue correspondent modules neither in the studies conducted with post-mortem brains nor in those using iPSC-derived cells. During the selection of the studies for our comparative analysis, among those that were re-analyzing data from other studies, we made the option to use only that with the largest number of ASD datasets, to avoid redundant analysis, and the study published by Gandal et al. [2] was the one that fulfilled this criteria at that time.

However, as well pointed out by Michael Lombardo [3], two previous studies, not included in our comparison, had identified modules with strong overlap to M_NPC_10-blue that were presented as dysregulated in ASD individuals: a study from this author [4], that re-analyzed the data from Voineagu et al. [5] and Gupta et al. [6] and a recent published paper from Gandal et al. [7], that re-analyzed the data published by Parikshak et al. [8] in conjunction with transcriptome data of other neuropsychiatric conditions. Considering the results found by these two studies, it is notable a replicable finding of dysregulation of this module of genes related to translation processes. As highlighted by Lombardo et al. [4], an expression relationship was found between this module and the module of synaptic genes and also, these two networks have an enrichment of protein–protein interaction, suggesting a regulatory relationship between them.

As thoroughly discussed by this author in his recent letter [3], all of this evidence supports an important role of genes involved in translation processes in ASD pathophysiology, not only of those well characterized ASD candidate genes such as *FMR1, TSC2, PTEN*, but also of the ribosomal protein genes that are the major components of the translation-related module dysregulated in ASD neuronal cells, which are less explored in literature. On the other hand, it is worthy to note that there is no evidence of its genetic association to ASD: all the studies that conducted this type of analysis failed to demonstrate enrichment of Sfari genes, common or rare variants in this network (geneM15 in Gandal et al. [7] and M_NPC_10-blue in Griesi-Oliveira et al. [1] M9 in Voineagu et al. [5], mod4 in Gupta et al. [6] CTX.M1 in Parikshak et al. [8] M14 in Parikshak et al. [9] saddle brown in Schafer et al. [10]—all these correspondence were based on our module overlap analysis depicted in Figure 5 in Griesi-Oliveira et al. [1]). Also, as reported by our group, a correspondent module to M_NPC_10-blue is preserved in iPSC-derived neurons in our study, however, this module is not dysregulated in this cell type. Based on these observations, it would be worthy to investigate possible mechanisms that are causing an expression dysregulation of this module in ASD neuronal cells, such as epigenetic mechanisms or a feed-back regulation occurring due to the dysregulation of the module of synaptic genes, since more robust genetic evidence points to this network as primarily impacted in ASD. This second hypothesis is supported by the expression and protein–protein interaction relationships between these two modules demonstrated by Lombardo et al. [4], and could explain their correlated dysregulation in neurons, although do not explain the expression dysregulation of M_NPC_10-blue in NPC. Another interesting question to be explored would be if the dysregulation of the module of translation found in post-mortem brain is actually happening in glial cells instead of in neurons, since in iPSC-derived neurons this was not evidenced [1, 10–12]. Although Gandal et al. [7] pointed out that this module is enriched by markers of excitatory neurons, there is robust evidence that this module is well preserved across different types of cells, such as NPC and even blood.

In summary, we agree with Dr Lombardo that evidence support that translation processes and ribosome proteins should be focused on the investigation of ASD pathophysiology, and particularly, we propose that the causes of dysregulation of this module should be explored from an epigenetic perspective and/or as a secondary change triggered by expression alteration of synaptic genes.
